# Micronutrient Deficiencies in the Era of Second-Generation Incretin-Based Therapies for Obesity

**DOI:** 10.3390/nu18040677

**Published:** 2026-02-19

**Authors:** Andrijana Koceva, Andrej Janež, Tajda Pečko, Mojca Jensterle

**Affiliations:** 1 Department of Endocrinology and Diabetology, University Medical Center Maribor, 2000 Maribor, Slovenia; 2 Faculty of Medicine, University of Maribor, 2000 Maribor, Slovenia; 3 Department of Endocrinology, Diabetes and Metabolic Diseases, University Medical Center Ljubljana, 1000 Ljubljana, Slovenia; 4 Faculty of Medicine, University of Ljubljana, 1000 Ljubljana, Slovenia; 5 Department of Nursing Care, University Medical Center Maribor, 2000 Maribor, Slovenia

**Keywords:** obesity, GLP-1-based therapy, incretin-based therapy, micronutrients, nutrition

## Abstract

Second-generation incretin-based therapies have transformed the pharmacological management of obesity by inducing substantial and sustained weight loss. The weight-reducing effects are primarily mediated through appetite suppression, reduced energy intake, and modulation of eating behavior. While therapeutically beneficial, these mechanisms may also influence dietary quality, micronutrient exposure, and overall nutritional status, particularly in individuals with obesity, a population already characterized by a high prevalence of baseline nutritional inadequacy. This narrative review is intended to inform clinicians, clinical nutrition specialists and researchers involved in obesity management by summarizing baseline micronutrient vulnerability in obesity, synthesizing available evidence on dietary intake, biochemical micronutrient status, and nutrition-related clinical outcomes during incretin-based therapy, discussing plausible mechanisms linking these therapies to micronutrient risk, and outlining approaches to risk-stratified nutritional monitoring in clinical practice.

## 1. Introduction

The rapid expansion of so-called second-generation incretin-based therapies has fundamentally transformed the pharmacological management of obesity. “Second-generation” refers to newer, higher-potency incretin-based agents with enhanced weight-loss efficacy, prolonged duration of action and broader metabolic effects compared with earlier therapies. These include the GLP-1 receptor agonist (GLP-1 RA) semaglutide and the dual GLP-1/glucose-dependent insulinotropic peptide (GIP) receptor agonist tirzepatide, which produce clinically meaningful and sustained weight loss, alongside improvements in glycemic control and cardiometabolic risk factors [1,2,3,4]. In phase 3 clinical trials, once-weekly semaglutide 2.4 mg resulted in a mean weight loss of approximately 14.9% over 68 weeks [1], while once-weekly tirzepatide achieved mean reductions of up to 20.9%, with one in three participants achieving ≥25% weight loss, closing the gap between previous obesity pharmacotherapy and metabolic surgery [3].

The weight-reducing effects of incretin-based therapy are primarily mediated through reductions in energy intake driven by enhanced satiety and reduced hunger. These effects are supported by gastrointestinal mechanisms, including delayed gastric emptying, increased gastric retention of solid meals and altered gut motility, which collectively promote earlier satiation and reduced meal size [5,6]. Beyond homeostatic appetite regulation, emerging evidence suggests that these agents modulate reward-related eating behaviors by influencing neural pathways involved in food motivation, cravings and hedonic eating [7,8]. These neuromodulatory effects may reduce the rewarding value of high-calorie foods and external food cues, contributing to sustained reductions in energy intake beyond the effects of satiety alone [7,8].

While their impact on appetite regulation, gastrointestinal function, and eating behavior underpins therapeutic efficacy, they may also have downstream consequences for nutritional status, particularly in individuals with obesity, a population already characterized by a high prevalence of baseline micronutrient inadequacy [9]. Despite the rapidly increasing use of incretin-based therapies in obesity management, their implications for micronutrient intake, biochemical micronutrient status, and nutrition-related clinical outcomes remain incompletely characterized. Most pivotal randomized controlled trials prioritize weight loss, glycemic outcomes, and cardiovascular endpoints, while systematic evaluation of dietary intake, dietary quality and micronutrient status is rarely incorporated [10].

This narrative review was informed by a targeted PubMed search for English-language human studies published between January 2010 and January 2026. The search was also complemented by manual screening of reference lists from key original studies, systematic reviews, and clinical nutrition guidelines. Search terms included incretin-based obesity pharmacotherapy (“GLP-1 receptor agonist”, “liraglutide”, “semaglutide”, “dual agonist”, “tirzepatide”) combined with nutrition-related outcomes (“micronutrient”, “vitamin”, “mineral”, “food intake”, “diet variety”, “diet quality”, “deficiency”). Priority was given to randomized controlled trials, observational studies, pharmacovigilance analysis, and mechanistic studies reporting dietary, biochemical, and nutrition-related outcomes. We aimed to summarize micronutrient vulnerabilities commonly present in obesity, evaluate existing evidence on dietary intake, biochemical micronutrient status and nutrition-related clinical outcomes during incretin-based therapy, discuss plausible mechanisms linking incretin pharmacotherapy to micronutrient risk, and outline implications for nutritional monitoring in clinical practice.

Recent reviews have discussed nutritional considerations during incretin-based therapy, primarily by extrapolating monitoring principles from metabolic surgery to pharmacological obesity treatment, providing valuable conceptual frameworks that highlight the importance of nutritional surveillance in the era of incretin-based therapies [11]. In contrast, the present review specifically addresses baseline micronutrient vulnerability in obesity, synthesizes emerging signals of micronutrient depletion and deficiency during incretin-based therapy, and integrates mechanistic pathways linking appetite suppression, dietary pattern changes, and gastrointestinal physiology and micronutrient handling. By integrating mechanistic pathways with real-world and biochemical signals, this review aims to inform a risk-stratified, obesity-specific approach to micronutrient monitoring that extends beyond surgical paradigms.

## 2. Baseline Micronutrient Vulnerability in Obesity

Micronutrients represent essential vitamins and trace elements required in small amounts for normal metabolic, neurological, immune and musculoskeletal function [12]. Micronutrient inadequacy is frequently observed in individuals with obesity despite excess energy intake, a phenomenon often described as being “overfed but undernourished” [9]. This vulnerability reflects a combination of suboptimal dietary quality, obesity-related inflammation, altered nutrient distribution and metabolism, and increased micronutrient requirements [13]. Understanding this baseline risk is essential when evaluating micronutrient signals observed during incretin-based therapy, as treatment-related reductions in intake may exacerbate pre-existing depletions rather than induce de novo deficiencies.

Dietary patterns commonly associated with obesity are characterized by higher consumption of energy-dense, ultra-processed foods and lower intake of fruits and vegetables, whole grains, and dairy products, resulting in reduced micronutrient density. In a cohort of individuals with morbid obesity, 40% of individuals were found to have three or more micronutrient deficiencies prior to any intervention [14].

Obesity is associated with an increased volume of distribution for lipophilic substances, including fat-soluble vitamins A, D, E and K [13]. As adipose tissue mass increases, a greater proportion of these vitamins is sequestered in fat stores, resulting in lower circulating concentrations despite potentially adequate total body content [13].

Vitamin D deficiency or insufficiency is the most consistently reported micronutrient abnormality in obesity. Vitamin D deficiency is seen at a higher prevalence in individuals with obesity, particularly in cohorts with severe obesity, compared to normal-weight and overweight populations [15,16,17,18]. Mechanistically, reduced vitamin D status in obesity is partly explained by decreased vitamin D bioavailability due to volumetric dilution and adipose tissue sequestration, leading to lower circulating 25-hydroxyvitamin D concentrations for a given intake or sun exposure [19]. Additionally, increased catabolism of vitamin D metabolites in adipose tissue and impaired hepatic conversion in the presence of metabolic dysfunction-associated steatotic liver disease also contribute to lower vitamin D levels [13].

Lower serum carotenoid concentrations and increased risk for vitamin A deficiency have also been reported in individuals with obesity [20,21,22]. These alterations are attributed to increased oxidative stress, inflammation and altered metabolism in obesity. Importantly, lower carotenoid levels have been observed even when dietary intake does not differ, suggesting that altered utilization rather than intake alone is the cause [13].

Evidence regarding vitamin B status in obesity is more heterogeneous, inconsistent, and largely observational, with some studies showing lower concentrations of vitamin B1 (thiamine), B6 (pyridoxine), B9 (folic acid) or B12 (cobalamin), while others show no significant deficiencies [18,23,24,25]. However, increased metabolic demand, dietary quality, and concomitant medication use, such as metformin in type 2 diabetes, may further contribute to vulnerability in certain subgroups [13]. Individuals with overweight or obesity also have a higher prevalence of vitamin C deficiency, a water-soluble antioxidant vitamin often found in fruits and vegetables. This deficiency likely reflects a lower intake of fruits and vegetables and increased oxidative stress associated with obesity [13,23].

Iron levels and iron homeostasis are frequently altered in obesity, likely due to chronic low-grade inflammation [18,26]. Elevated inflammatory signaling increases hepatic hepcidin expression, which reduces intestinal iron absorption and mobilization from stores, leading to functional iron deficiency even when total body stores are not severely depleted [26].

Obesity is further associated with an altered status of several trace elements, including zinc, magnesium, manganese, chromium and selenium [13]. Zinc deficiency is particularly relevant given its role in insulin synthesis, storage, and signal transduction. Lower serum zinc concentrations have been consistently reported in individuals with obesity, reflecting both increased metabolic demand and suboptimal dietary intake [16,27,28].

Table 1 summarizes common baseline micronutrient vulnerabilities in obesity and their respective key mechanisms.

## 3. Evidence for Micronutrient-Related Outcomes During Incretin-Based Therapy

Direct evidence linking incretin-based therapy to micronutrient deficiency remains limited and current evidence is derived primarily from real-world claims data, pharmacovigilance analyses, dietary intake studies and a small number of observational or mechanistic investigations [29,30,31,32,33].

### 3.1. Real-World Signals of Nutritional Deficiency and Related Complications

The strongest large-scale signal for nutrition-related outcomes comes from a retrospective claims study including 461,382 adults prescribed GLP-1 receptor agonists. By using diagnostic codes at 6 and 12 months after treatment initiation, this study identified nutritional deficiencies and deficiency-related complications in 12.7% of patients at 6 months and 22.4% at 12 months [29]. Recorded diagnoses included vitamin deficiencies, mineral deficiencies, nutritional anemia, iron deficiency anemia, thiamine deficiency and volume depletion. Vitamin deficiency codes were most frequently recorded, driven primarily by vitamin D deficiency (7.5% at 6 months and 13.6% at 12 months), followed by other vitamin B deficiencies (1.3% at 6 months and 2.6% at 12 months), thiamine deficiency (0.1% at 12 months), mineral deficiency (0.4% at 6 months and 0.8% at 12 months), volume depletion (1.8% at 6 months and 3.5% at 12 months) and iron deficiency anemia (1.6% at 6 months and 3.2% at 12 months) [29].

Importantly, deficiencies were more commonly documented among individuals who had a dietitian visit within 6 months of initiating incretin-based therapy, consistent with probable detection or surveillance bias rather than direct pharmacologic causation [29]. Although these findings do not establish the incidence of biochemical deficiency, they provide clinically relevant signals that nutrition-related complications are being identified in routine care among patients receiving incretin-based therapy.

### 3.2. Pharmacovigilance Evidence: Dehydration as Nutritional Signal

Post-marketing pharmacovigilance analysis provides complementary evidence by capturing spontaneously reported adverse events. A disproportionality analysis of the FDA Adverse Event Reporting System (FAERS) identified dehydration as a prominent and consistent signal among multiple agents [30]. Dehydration events were frequently reported early after treatment initiation, often within the first month. While dehydration is not a micronutrient deficiency, it represents a clinically meaningful complication that may reflect reduced fluid intake, gastrointestinal adverse events or concurrent reductions in food intake, underscoring the relevance of nutritional monitoring during early phases of incretin therapy, particularly during dose escalation [30].

### 3.3. Dietary Intake Patterns and Risk of Micronutrient Inadequacy

Beyond diagnostic signals, dietary intake studies provide additional context. A cross-sectional study using 3-day dietary records in 69 adults treated with incretin-based therapy, mostly semaglutide (53.6%) or tirzepatide (33.3%) for at least a month, demonstrated widespread nutrient inadequacies, such as inadequate average intakes of multiple micronutrients, including calcium, iron, magnesium, potassium, vitamins A, C, D, E, K, and choline, relative to dietary reference intakes [31]. Participants also overconsumed calories from fat and saturated fat, while showing inadequate intake of key food groups, including vegetables, grains, fruit, and dairy products. Dietary fiber intake was also inadequate, and protein intake was also below levels commonly recommended for lean mass preservation during weight loss, with only 10% meeting a protein intake of at least 1.6 g/kg/day [31].

Another cross-sectional study reported suboptimal diet quality, with low Healthy Eating Index scores and skewed meal patterns, with the largest proportion of energy and protein consumed at dinner, potentially affecting micronutrient distribution associated with protein intake and meal timing. The group also found low intakes of fruit, vegetables, whole grains, dairy and plant/seafood proteins. Since these food groups are primary sources of key vitamins and minerals, a reduced diet quality may increase the risk of micronutrient inadequacy [34].

### 3.4. Biochemical and Functional Evidence of Micronutrient Alterations

Biochemical data remains scarce. In a retrospective study, Japanese patients with obesity and type 2 diabetes received semaglutide at least 12 months after prior sleeve gastrectomy. Over 12 months of semaglutide treatment, the group observed significant declines in vitamin B12 and zinc concentrations, accompanied by a macronutrient dietary shift from protein to carbohydrate compared with post-surgery patterns [32].

While most available literature on incretin-based therapy describes signals consistent with micronutrient depletion rather than overt deficiency, rare cases of clinically significant micronutrient deficiency have been reported [35]. Notably, Wernicke encephalopathy has been reported in patients receiving incretin-based therapy in the context of prolonged vomiting and reduced food intake [35]. A recent pharmacovigilance analysis identified 15 cases of GLP-1 RA-associated Wernicke encephalopathy, most commonly reported with semaglutide and tirzepatide, with increased reporting odds compared with other medications, highlighting the need for clinical vigilance in patients with severe gastrointestinal symptoms and prolonged poor intake [36].

A pilot study assessing intestinal iron absorption using an oral iron absorption test before and after initiation of semaglutide demonstrated attenuated intestinal iron absorption, with a median reduction of 13% after 10 weeks of treatment [33]. Additionally, 17.6% of participants experienced a ≥30% reduction from baseline. This diminished absorption is likely influenced by semaglutide’s effects on gastric emptying and gut motility and may contribute to iron deficiency or anemia, especially in susceptible individuals with pre-existing low iron stores. While long-term iron status and anemia outcomes were not assessed, this study demonstrates that incretin-based therapies may influence micronutrient handling through mechanisms beyond reduced intake alone [33].

Table 2 summarizes available evidence linking incretin-based therapy to micronutrient-related outcomes.

## 4. Mechanisms Linking Incretin-Based Therapy to Micronutrient Risk

Incretin-based therapies may influence micronutrient status through several overlapping and interacting pathways. These include reductions in total food intake and absolute micronutrient exposure, changes in dietary patterns and food-group selection, and gastrointestinal effects that may alter nutrient tolerance, timing and absorption.

### 4.1. Reduced Food Intake and Absolute Micronutrient Exposure

The most consistent and well-documented mechanism is a reduction in total energy intake. Incretin-based therapies reduce ad libitum intake primarily by enhancing satiety and reducing hunger. At the central level, GLP-1 receptor agonists act on key hypothalamic and brainstem nuclei involved in energy homeostasis. These include the arcuate nucleus, where GLP-1 signaling enhances anorexigenic pro-opiomelanocortin (POMC) neuron activity and suppresses orexigenic neuropeptide Y/agouti-related peptide (NPY/AgRP) pathways, thereby reducing hunger. Additional action within the paraventricular nucleus integrates satiety signals, while engagement of the nucleus tractus solitarious in the brainstem amplifies vagal afferent input from the gastrointestinal tract, reinforcing meal termination [37]. Across multiple crossover and parallel-group trials, liraglutide, oral semaglutide, once-weekly semaglutide and tirzepatide have been shown to reduce ad libitum energy intake by approximately 16–39%, with reductions occurring across meals and snack occasions [38,39,40,41,42]. These reductions in energy intake are accompanied by consistent changes in subjective appetite regulation, including lower hunger ratings, reduced prospective food consumption, and increased satiety and fullness, as assessed by validated visual analog scales and appetite questionnaires [38,39,40,42,43].

Beyond homeostatic feeding, GLP-1 receptor agonists also influence reward-related regions, including mesolimbic pathways involving the ventral tegmental area and nucleus accumbens, with several studies also demonstrating improvements in appetite control and eating behavior, including reductions in craving intensity or frequency, decreased food preoccupation (often captured as food noise) and reduced binge-eating episodes, reflecting dampened motivation to eat in response to external food cues rather than metabolic need [7,37,43,44]. Together, lower homeostatic hunger drive and reduced hedonic responding can decrease eating opportunities and total food volume even beyond intentional calorie restriction.

Caloric restriction and reduced food volume are well-recognized correlates of lower micronutrient intake [45]. If reductions in intake are not accompanied by deliberate optimization of diet quality, absolute intake of vitamins and minerals may decline proportionally, especially when daily energy intake falls below 1200 kcal for females or 1800 kcal for males [46]. Earlier satiety and smaller meal sizes may also reduce consumption of micronutrient-dense foods, while fewer eating occasions can further limit total micronutrient exposure [39,40]. Since micronutrient intake depends largely on food volume and dietary variety rather than energy alone, sustained reductions in intake may increase the risk of micronutrient depletion even when macronutrient requirements are met [46,47].

Lastly, reductions in total energy intake during incretin-based therapy are frequently accompanied by changes in macronutrient distribution, particularly a reduction in absolute protein intake, which may be insufficient to support lean mass preservation during weight loss if not actively addressed [31,34,46]. Lean body mass is increasingly recognized as a key determinant of nutritional resilience, serving as a functional reservoir for metabolic substrates and micronutrient-dependent processes [12], and observational data demonstrate positive associations between lean mass and micronutrients, such as selenium, zinc, vitamin B12, iron and vitamin D, underscoring the interplay between body composition, macronutrient adequacy and micronutrient status in the context of weight loss and reduced intake, including pharmacologically induced weight loss [48].

Direct comparative data evaluating differences between micronutrient outcomes between mono-GLP-1 receptor agonists and dual GLP-1/GIP receptor agonists are currently lacking. Dual agonists generally produce greater weight loss [1,3], which may theoretically lead to larger reductions in energy intake and amplify intake-mediated micronutrient risk by more pronounced reductions in total food volume and dietary variety. However, available evidence does not allow conclusions on differential micronutrient risk by agonist class, which represents an important evidence gap requiring prospective investigation.

### 4.2. Dietary Pattern Changes and Food-Group Displacement

In addition to reducing total intake, incretin-based therapies may influence what foods are consumed. Mechanistic studies consistently demonstrate reduced hunger or cravings and shifts away from high-fat, energy-dense foods. While reduced hunger and improved control of eating are metabolically favorable, these changes may translate into fewer eating opportunities and less variety if not actively managed [39,40]. A recent secondary analysis of a randomized trial showed that tirzepatide significantly reduced preferences for foods high in fat and simple sugars, as well as reduced craving scores for sweets, carbohydrates and fast-food fats [49]. Similarly, studies with oral semaglutide have reported decreased total energy intake, with the greatest relative reduction in carbohydrate intake, accompanied by diminished cravings and a reduced desire for sweets [50].

Although these shifts may reduce intake of highly processed foods, they may also displace food groups that are important sources of specific micronutrients, such as iron, zinc, vitamin B12 (animal-source foods), calcium (dairy), or folate and vitamin C (fruits and vegetables), particularly when meals are smaller, fewer and less varied. This reduced dietary diversity has consistently been associated with lower micronutrient adequacy across populations [51,52]. Mechanistically, observed reductions in fruits, vegetables, and whole-grain intake may plausibly reflect a combination of earlier satiation and smaller meal size, avoidance of foods that exacerbate nausea or dyspepsia, and centrally mediated shifts in reward-related eating that reduce cravings, hedonic drive and preference for sweet-tasting foods [7,39,40,49,50].

### 4.3. Gastrointestinal Physiology and Adverse Events

Symptoms commonly labeled as gastrointestinal adverse events, such as nausea, vomiting, and diarrhea, are largely mediated via central nervous system pathways despite manifesting peripherally and directly affecting food tolerance and intake. These adverse events are relatively common during incretin-based therapy, particularly during dose escalation, are usually transient and mild-to-moderate in severity, and may temporarily disrupt habitual eating patterns, reduce food tolerance and decrease both food and fluid intake [30]. In cases of prolonged gastrointestinal intolerance, these effects may increase the risk of dehydration and micronutrient depletion.

In addition, incretin-based therapies are also associated with delayed gastric emptying of solid meals, increased gastric retention, and prolonged half-emptying time, reflecting altered gastrointestinal transit [5,6]. While slowing of gastric emptying contributes to satiety and glycemic control, it may also influence the timing and efficiency of nutrient delivery to absorptive sites in the small intestine. Emerging mechanistic data suggest that these effects may be relevant for specific nutrients, such as iron, for which reduced absorption has been demonstrated following initiation of semaglutide [33].

Pancreatic exocrine insufficiency has been proposed as a theoretical contributor to nutrient malabsorption during incretin-based therapy. However, available human data do not support this mechanism. In a randomized study comparing short-acting and long-acting GLP-1 receptor agonists, fecal pancreatic elastase concentrations and other markers of exocrine pancreatic function did not deteriorate following treatment, and no evidence of fat malabsorption was observed [53]. These findings suggest that pancreatic exocrine insufficiency is unlikely to contribute meaningfully to micronutrient risk.

### 4.4. Interaction with Concomitant Pharmacotherapy and Comorbidities

In addition to direct effects on appetite, dietary intake and gastrointestinal physiology, micronutrient status during incretin-based therapies may also be influenced by concomitant pharmacological treatment commonly used in populations with obesity. Metformin, frequently prescribed in individuals with type 2 diabetes and polycystic ovary syndrome, has been consistently associated with reduced vitamin B12 absorption and lower circulating levels, particularly in long-term use [12,54]. Given the high prevalence of combined metformin and incretin-based therapy in clinical practice, an integrated nutritional assessment is necessary.

**Figure 1:**
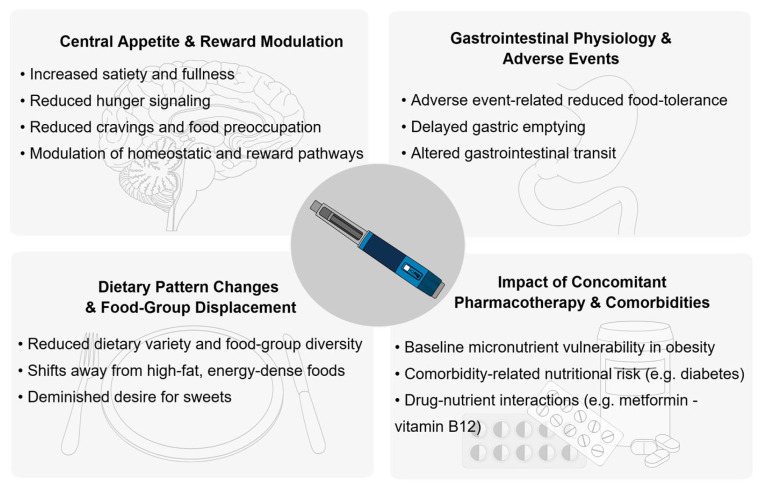
Key mechanisms by which incretin-based therapy impacts micronutrient status.

Figure 1 summarizes key mechanisms by which incretin-based therapy impacts micronutrient status.

## 5. Nutritional Monitoring During Incretin-Based Therapy

The convergence of baseline micronutrient vulnerability in obesity and the appetite-suppressing effects of incretin-based therapy raises important clinical considerations for nutritional monitoring. Recent obesity-specific nutrition frameworks emphasize that pharmacological obesity treatments, including incretin-based therapies, should be accompanied by structured dietary strategies to preserve nutrient adequacy and lean mass, particularly in the context of pharmacologically induced reductions in energy intake [20]. Comparisons are frequently drawn between incretin-based pharmacotherapy and metabolic surgery due to the magnitude of weight loss achieved; nevertheless, important distinctions must be recognized. Metabolic surgery is associated with well-established, procedure-specific risks of micronutrient deficiencies driven by anatomical alterations and malabsorption, resulting in predictable deficiencies such as iron, vitamin B12, calcium, and fat-soluble vitamins [11]. In contrast, observed signals in incretin-based therapy are likely to be driven by reduced dietary intake, altered food selection, and, in selected cases, delayed nutrient absorption rather than true malabsorption [10,29,33]. Thus, while metabolic surgery provides a useful conceptual framework for anticipating nutritional risk during substantial weight loss, direct extrapolation of deficiency prevalence, severity, and supplementation strategies to incretin-based therapy is not appropriate.

Although current evidence does not support a blanket micronutrient supplementation for all patients receiving incretin-based therapy, it does support a risk-stratified approach to nutritional assessment and follow-up, consistent with established clinical nutrition guidance [12]. Micronutrients most frequently identified in real-world data and dietary intake studies overlap with those highlighted for in the recent expert recommendations outlining nutritional priorities to support incretin-based therapy in obesity, which emphasize that pharmacologically induced reductions in energy intake increase the risk of insufficient intake of several key micronutrients, particularly vitamin D, iron, vitamin B12, calcium, and magnesium, unless diet quality is actively maintained [46].

Importantly, nutritional monitoring during incretin-based therapy should distinguish between dietary intake inadequacy, micronutrient depletion and overt deficiency [12]. Initial assessment should prioritize dietary quality, food-group representation, gastrointestinal tolerance and weight loss trajectory, with laboratory testing reserved for individuals with clinical risk factors, symptoms suggestive of deficiency, or prolonged reductions in intake, consistent with clinical nutrition guidance [12,20,46].

Individuals at higher nutritional risk include those experiencing rapid or pronounced weight loss, persistent gastrointestinal adverse effects, restrictive dietary patterns or reduced dietary variety. Patients with pre-existing micronutrient insufficiency, severe obesity or a history of metabolic surgery represent particularly vulnerable subgroups [13,14]. Older age is another well-recognized risk factor for nutritional vulnerability, characterized by higher rates of inadequate energy, protein and micronutrient intakes, as well as increased prevalence of sarcopenia and functional decline [55,56]. Age-related changes intersect with pharmacologically induced reductions in appetite and energy intake, warranting enhanced nutritional monitoring and tailored dietary strategies in these individuals. Real-world data suggest that nutrition-related diagnoses are more frequently identified among individuals who interact with dietitians, underscoring the importance of active clinical surveillance and nutritional assessment rather than passive observation alone [29].

Given their high baseline prevalence in obesity and consistent emergence as signals during therapy, vitamin D and iron status warrant particular clinical attention. Baseline evaluation may include a structured dietary assessment focused on nutrient density and food-group representation [29]. The early treatment phase, particularly during dose escalation, may represent a critical window for identifying nutritional risk, while progressive accumulation of nutrition-related diagnoses over time suggests that ongoing surveillance may be more appropriate than one-time assessment [29]. In individuals at increased nutritional risk, targeted laboratory assessment may include serum ferritin and iron indices, 25-hydroxyvitamin D, vitamin B12, and, where clinically indicated, folate and zinc levels. Baseline evaluation should be performed prior to or shortly after treatment initiation in high-risk patients, with repeat assessment at approximately 6–12 months or earlier if symptoms, rapid weight loss, or prolonged gastrointestinal intolerance develop.

From a dietary perspective, nutritional management during incretin-based therapy should prioritize ensuring nutrient adequacy within a lower-energy dietary pattern, rather than caloric restriction alone. Consensus recommendations emphasize the importance of maintaining diet quality by ensuring adequate intake of fruits, vegetables, whole grains, protein-rich foods, nuts, and seeds, as well as adopting healthy eating habits with regular small meals and adequate hydration [46]. During active weight reduction, higher protein targets are often recommended, with absolute daily targets ranging from 80 to 120 g or, if body composition data is available, 1.5 g per kg of fat-free mass per day [46]. Practical dietary counseling may include prioritizing protein-rich foods at each meal, emphasizing nutrient-dense foods early in the meal when appetite is the greatest, maintaining regular meal patterns and ensuring adequate fluid intake. Physical activity, particularly resistance and mixed-mode exercise, also plays a critical role in preserving lean body mass during weight loss and may mitigate reductions in appetite and nutrient intake [46].

To mitigate gastrointestinal adverse effects commonly observed during incretin-based therapy, such as nausea, vomiting, or dyspepsia, supportive dietary strategies are commonly recommended. These include consuming smaller, more frequent meals, stopping intake before reaching fullness, and avoiding high-fat, spicy foods, alcoholic beverages, and carbonated beverages [20]. In individuals with constipation, attention to adequate fluid intake and gradual dietary fiber optimization may be beneficial. When non-pharmacological measures are insufficient, short-term use of acid-suppressive therapy (proton pump inhibitors or H2 receptor blockers) or laxatives may be considered on an individual basis [20].

## 6. Limitations and Future Directions

This review has several limitations inherent to its narrative design. The available evidence is dominated by observational studies, retrospective analysis, dietary assessments, and pharmacovigilance data, with a notable absence of randomized controlled trials specifically designed to evaluate micronutrient outcomes during incretin-based therapies. Consequently, causal influences remain limited and the prevalence of biochemical deficiency may be underestimated. In addition, heterogeneity in study populations, treatment durations, and outcome definitions restrict direct comparisons across studies. These limitations further emphasize the need for well-designed prospective studies to inform evidence-based nutritional monitoring strategies. Future research should aim to characterize temporal changes in micronutrient status across different treatment phases, identify patient subgroups at higher nutritional risk, and determine whether observed micronutrient depletions translate to clinically meaningful outcomes. Cooperative studies examining monoagonists and dual agonist therapies are also warranted, as well as pragmatic nutritional monitoring strategies in clinical practice.

## 7. Conclusions

To conclude, existing evidence suggests that incretin-based therapy may affect micronutrient status through reduced dietary intake, altered dietary patterns, gastrointestinal effects, and, in selected cases, impaired nutrient absorption. However, current evidence is characterized by indirect signals rather than definite causal evidence and the true incidence rates of biochemical micronutrient deficiency during incretin-based therapy remain unknown. These findings highlight the need for prospective, longitudinal studies that systematically integrate dietary assessment, standardized biochemical micronutrient panels and body composition measures in patients treated with incretin-based therapies. A risk-stratified approach, prioritizing dietary quality, muscle mass preservation, and targeted laboratory assessment in high-risk individuals, offers a pragmatic framework to mitigate nutritional risk while preserving therapeutic efficacy.

## Figures and Tables

**Table 1:** Common baseline micronutrient vulnerabilities in obesity and plausible mechanisms.

Micronutrient	Typical Findings in Obesity	Key Mechanistic Drivers	References
Vitamin D	Lower circulating 25(OH)D	Volumetric dilution/adipose sequestration. Altered metabolism in obesity.	[15,16,17,19]
Vitamin A/ carotenoids	Lower serum carotenoids	Oxidative stress/inflammation. Altered metabolism and distribution.	[17,21,22]
B vitamins (B1, B6, folate, B12)	Variable lower values depending on population and concomitant medications	Diet quality. Increased metabolic demand. Concomitant medications.	[13,16,18,23,24,25]
Vitamin C	Lower intake/status more frequent	Low fruit/vegetable intake. Oxidative stress.	[13]
Iron	Functional iron deficiency tendency	Low-grade inflammation, higher hepcidin, and lower absorption and iron mobilization.	[16,17,18,26]
Zinc	Lower serum zinc reported in obesity	Lower intake/diet quality. Increased requirement/inflammation.	[16,27]
Magnesium	Often lower intake/status	Diet quality.	[13,17]

**Table 2:** Evidence linking incretin-based therapy to micronutrient-related outcomes.

Population and Design	Therapy	Outcome Assessed	Key Findings	Limitations
Adults with type 2 diabetes (large real-world cohort; N = 461,382) [29]	GLP-1 RAs (class level)	ICD-coded nutritional deficiencies/complications	Higher incidence of ICD-coded diagnoses of vitamin D deficiency, B vitamin deficiency, iron deficiency, anemia, mineral deficiency and volume depletion.	Strongest signal dataset, but diagnosis code-based. No biochemical assessment. Detection or surveillance bias.
Adults using GLP-1 and dual GLP-1/GIP therapies (cross-sectional dietary assessment, N = 69) with 3-day records [31]	Semaglutide (53.6%) Tirzepatide (33.3%) Dulaglutide (11.6%) Liraglutide (1.4%)	3-day food records; MyPlate food group servings	Multiple nutrient intakes below daily reference intakes (fiber, calcium, iron, magnesium, potassium, vitamins A, C, D, E, K and choline). Lower intake in fruit, vegetables, grains and dairy. Excess intake in total and saturated fat. Protein intake ≥1.6 g/kg/day only in 10%.	Cross-sectional study. No biochemical assessment. No baseline comparison.
Post-sleeve gastrectomy patients with obesity and type 2 diabetes (retrospective single-center cohort, N = 29) [32]	Semaglutide	Serial serum nutritional metrics and reported macronutrient shift	Lower levels of B12 and zinc concentration.	Small sample. Selected high-risk population (post-metabolic surgery).
Type 2 diabetes (mechanistic study) [33]	Semaglutide	Iron	Reduction in iron absorption.	Short duration.
Case reports of GLP-1 RA-associated events (real-world pharmacovigilance and case literature) [35]	Semaglutide Tirzepatide	Clinically overt thiamine deficiency	15 reported Wernicke encephalopathy cases in the context of prolonged vomiting, gastrointestinal side-effects and reduced intake.	Rare but clinically important outcome/signal. Causal attribution is limited.

## Data Availability

No new data were created or analyzed in this study. Data sharing is not applicable to this article.
